# Minimally Invasive Surgical Approaches for Bladder Outlet Obstruction Within an Office Environment: A Comprehensive Literature Review

**DOI:** 10.5152/tud.2025.25034

**Published:** 2025-06-04

**Authors:** Jack Harkins, Ali Talyshinskii, B. K. Somani, Feras Al Jaafari

**Affiliations:** 1 University of St Andrews, St Andrews, Scotland; 2 Ferghana Medical Institute of Public Health, Fergana, Ferghana Province, Uzbekistan; 3 Department of Urology, University Hospital Southampton NHS Foundation Trust, Southampton, England

**Keywords:** BPH, iTIND, MIST, prostate, Rezum, UroLift

## Abstract

Benign prostatic hyperplasia (BPH) is a common urological disorder in aging men, leading to bladder outlet obstruction (BOO) and lower urinary tract symptoms (LUTS). Traditional surgical treatments are associated with hospitalization, general anesthesia, and potential sexual dysfunction. Minimally invasive surgical techniques (MISTs), such as Rezum, UroLift, and iTIND, offer office-based, local anesthesia options with fewer adverse events. This systematic review evaluates the efficacy, safety, and durability of these MIST procedures. A systematic search of OVID Medline and Embase was conducted in July 2024 using Boolean operators with terms related to BPH and MIST procedures. Inclusion criteria included English language studies using MIST with a minimum follow-up of 1 year. Data were extracted and analyzed, focusing on functional outcomes, retreatment rates, complications, and anesthesia use. Thirteen studies met inclusion criteria, with a total of 1,864 patients (Rezum 6 studies, 1,292 patients; UroLift 4 studies, 331 patients; and iTIND 3 studies, 241 patients). Rezum, UroLift, and iTIND all demonstrated significant improvements in Qmax and International Prostate Symptom Score (IPSS). Sexual function remained largely preserved. While retreatment rates varied, UroLift had the highest rate (mean: 6.4%). Procedural complications were generally mild, with acute urinary retention being the most frequent. Rezum, UroLift, and iTIND can be done safely and successfully as a day case procedure usually under local anesthetic. The short-term results are good for all of these procedures and improves objective outcomes as well as preserves or improves sexual function. However, further long-term randomized controlled trials comparing multiple treatment modalities are needed to refine patient selection and optimize outcomes.

## Introduction

Benign prostatic hyperplasia (BPH) is a prevalent urological disorder affecting a large proportion of men with an increased prevalence with age.^1^ Changes in prostate architecture lead to bladder outlet obstruction (BOO) and, eventually, lower urinary tract symptoms (LUTS). This has a significant impact on patients’ quality of life and overall health status and necessitates careful assessment and management by the urologists.^2^ Existing treatments for BPH include watchful waiting (including lifestyle changes and dietary optimization), medical therapy, and if these fail, surgical intervention.^3^,^4^ The key risk factor for BPH is growing age, which also increases the likelihood of comorbidities in patients, and precludes conventional minimally invasive therapies, necessitating the use of newer surgical options.^5^ Despite the active transition from prostate resection to enucleation-based methods, these interventions are also associated with the need for hospitalization, the use of general anesthesia, changes in sexual and ejaculatory function, and the likelihood of developing various intra- and postoperative complications, all of which have a significant impact on associated final treatment costs.^6^,^7^

Minimally invasive surgical techniques (MISTs) are a more novel method of treating BPH and include Rezum, UroLift, and iTIND, which are associated with the ability to use local anesthesia, in-office-based setting, minimal sexual function-related side effects, reduction in adverse events during or after surgery, and overall cost-effectiveness as a consequence.^8^,^9^ Despite the existence of a sufficient number of original studies and reviews on each of these strategies, there is a lack of a comprehensive review of all of these techniques in the context of being done as an office-based day case procedure. The purpose of this research is to conduct a systematic evaluation of the existing literature in order to assess current MIST procedures for office-based treatment of BPH using Rezum, UroLift, or iTIND.

## Materials and Methods

Search: In July 2024, the systematic search was conducted across several databases, including OVID Medline, and OVID Embase via Boolean operators with the use of the following terms: “BPH,” ”LUTS,” “BOO,” “Rezum,” “vapour thermal therapy,” “UroLift,” “Prostatic urethral lift therapy,” “Temporary Implantable Nitinol Device,” “iTIND,” and “outcomes.”

Inclusion criteria for studies:

The research question for this review was formulated using the Population, Intervention, Comparison, Outcome and Setting framework: Population—adult males with LUTS secondary to BPH; Intervention—Rezum, UroLift, and iTIND; Comparison—any other surgical procedure including Transurethral resection of the prostate (TURP), enucleation procedure or sham therapy; Outcome—functional and personal outcomes. This framework was also used for the formation of the inclusion and exclusion criteria used for paper selection:

- Studies including adult males with LUTS related to BPH only.
- Use of Rezum, UroLift, and iTIND.
- Description of baseline and post-treatment Qmax and IPSS score.
- At least 1-year follow-up.
- Description of number of patients recruited.
- Cohort studies and randomized clinical trials.
- Full text accessible papers written in English.

To better cover literature, sexual outcomes and use of local anesthesia were also investigated, however, data on them was not strict inclusion criteria.

Exclusion criteria for studies:

- Studies including adult males with LUTS not related to BPH.
- Use of other modalities.
- The absence of details on baseline and post-treatment Qmax and IPSS score.
- Follow-up for less than 1 year.
- Abstracts and alternate study designs.

Studies process: Two reviewers independently identified all papers. All studies fitting inclusion criteria were selected for full review. If there was disagreement or discrepancy, the senior author (F.A.) made the final decision.

Data extraction and analysis: Studies were systematically reviewed, and information was extracted that was related to the study design, follow-up length, number of patients, retreatment rate and complications rates stratified according to Clavien-Dindo nomenclature, percentage of local anesthetic use. This review aims to analyse the use of minimally invasive treatment of male LUTS, secondary to BPH. To achieve this, an important criterion for article selection was the use of IPSS and Qmax scores when measuring patient outcomes.^10^ Alternative patient outcomes including sexual outcomes such as International Index of Erectile Function (IIEF) scores are also noted where available. Studies were systematically analysed using the Critical Appraisal Skills Programme checklists designed for either cohort studies or randomized controlled trials.^11^ Presented review was reported according to the Preferred Reporting Items for Systematic Reviews and Meta-Analysis (PRISMA) guidelines. The PRISMA Checklist for the process was followed.

## Results

**Figure 1.**
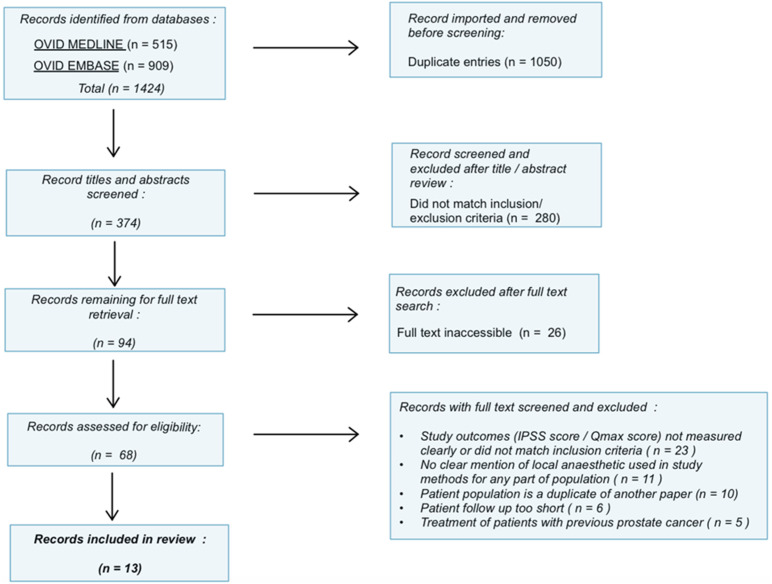
Preferred Reporting Items for Systematic Reviews and Meta-Analysis flowchart of the included studies.

Among 1424 studies 13 fitted inclusion criteria and were included in final review (Figure 1). Of these, 10 were observational cohort studies and 3 were randomized controlled trials (Tables 1 and 2).

### Rezum

Six studies investigated the Rezum procedure for patients with BPH. A total of 1292 patients were reported with individual studies reporting on 65-352 patients. There was only 1 randomized controlled trial (RCT), with follow-up data across studies of between 1 and 3 years. Across studies, the mean baseline IPSS varied from 18.0-22.7, and the mean Qmax varied from 7.9 to 10.4 mL/s. The use of local anesthesia (LA) was reported across 4 studies and ranged from 11.9% to 20.7% (Table 3). The studies included patients with a median lobe and prostate volumes up to 160 mL. The average procedural time varied between 4.8 and 12 minutes and was documented in 3 studies. Serious adverse events (Clavien-Dindo III/IV) were reported in 1 study in 1.7% of patients. One study found significant improvement in sexual outcomes, with retreatment rates of 1.5%-5.7% across studies (Table 4).

**Figure 2.**
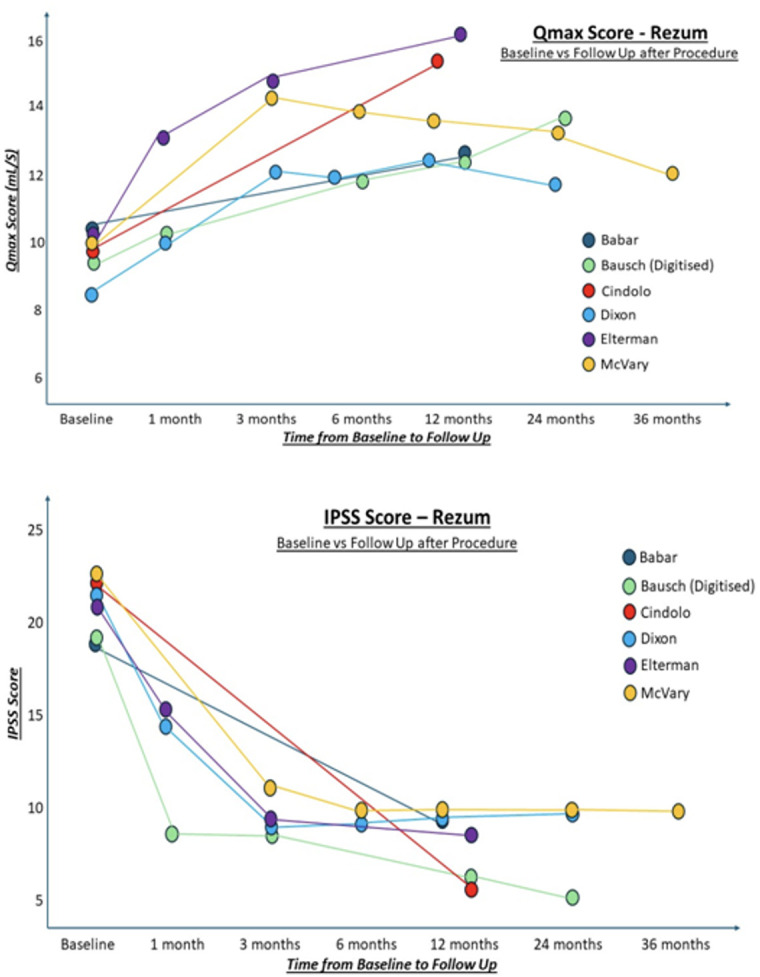
Rezum IPSS and Qmax across studies.

Dixon et al^12^ followed the outcomes of the Rezum procedure over 2 years for 65 recruited patients and found it safe and effective, with improvements in IPSS (−55.8%) and Qmax (+44.6%). Babar et al^13^ retrospectively analyzed a cohort of patients with outcomes and found that at 12 months, 80%, 87.5%, and 66% of patients in the mild, moderate, and severe LUTS cohorts discontinued their medications for BPH, respectively. Interestingly, no significant difference was found between those treated under general anesthesia (GA) and prostatic block. Bausch et al^14^ presented a real-world, longitudinal, “pragmatic” cohort study focused on functional and personal outcomes and found that Rezum provided significant improvements in IPSS (−65.7%) and Qmax (+66.7%).^14^ Cindolo et al^15^ presented a retrospective, multicentre cohort study measuring functional and personal outcomes of 352 Italian patients and also found significant improvement in both IPSS and Qmax.^15^ Elterman et al^16^ performed a large multicentre study including patients with 20-160 mL prostate volume, where 55% among latter had a median lobe present on cystoscopic examination. It showed that Rezum resulted in significant improvement in IPSS and Qmax, however, without change in IIEF scores. McVary et al^17^ performed an RCT to follow the outcomes of 197 patients who were double-blind randomized 2:1 for the Rezum procedure against a sham therapy including patients both with prostate volume up to 80 mL and with a median lobe. The Rezum procedure in this study was deemed safe and effective, with improvements in IPSS (−50.0%) and Qmax (+39.0%). The authors found a decrease of up to 3% in IIEF. The dynamics of IPSS and Qmax changes during follow-up across studies are presented in Figure 2.

### UroLift

Four studies investigated the UroLift procedure for BPH. A total of 331 patients were reported with individual studies reporting on 53-137 patients. Across studies, the mean baseline IPSS varied from 20.82 to 23.8, and the mean Qmax varied from 6.8 to 11.24 mL/s. The use of local anesthesia (LA) across 3 studies was above 95% (Table 3). All studies excluded cases with obstructive median lobe and included patients with prostate volumes up to 111 mL. The average procedure length varied from 16.8 to 66.14 minutes. Serious complications were reported in 1 study and were seen in 1% of patients. The retreatment rate varied from 0% to 12.8%. Significant improvement in sexual outcomes was found in 1 study (Table 4).

**Figure 3.**
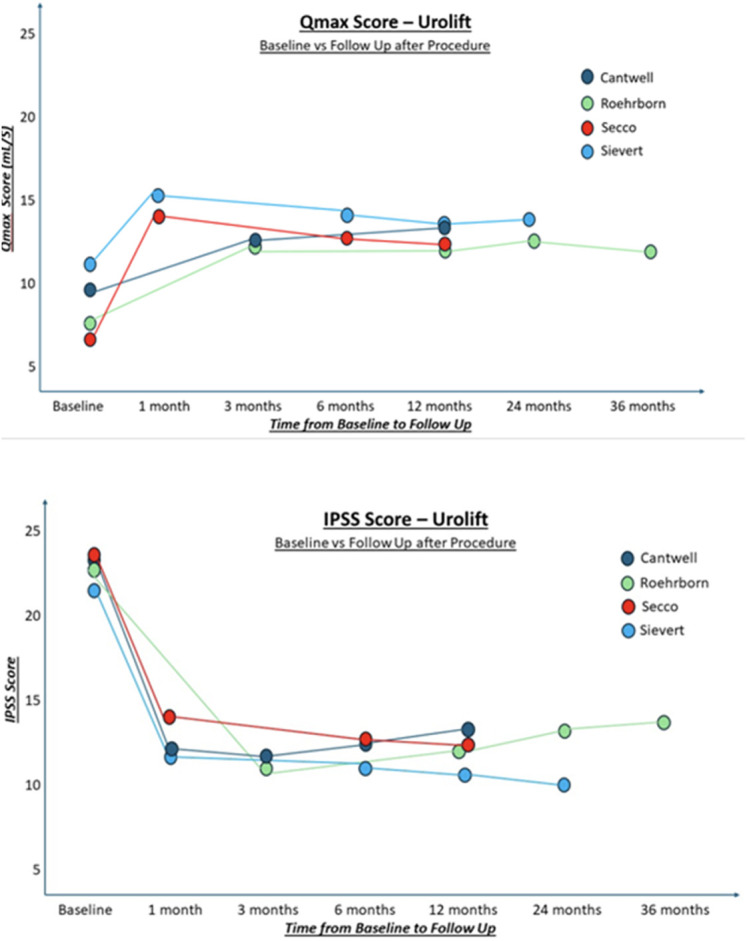
UroLift IPSS and Qmax across studies.

Cantwell et al^18^ followed the outcomes of 53 patients who were treated with UroLift after being the subjects of an initial sham procedure in a blinded pivotal RCT.^18^ The procedure was deemed safe and effective, with improvements in IPSS (−37.0%) and Qmax (+35.0%). Roehrborn et al^19^ performed a multicentre randomized, sham-controlled “pivotal” study to follow the outcomes of 140 patients. The study showed safety with improvements in IPSS (−41.0%) and Qmax (+53.1%). Sievert et al^20^ analyzed the effectiveness of UroLift among 86 patients from 5 centres and found improvements in IPSS (−51.15%) and Qmax (+53.1%). Secco et al^21^ reported a dual-centre, retrospective cohort study to follow the outcomes of 55 patients who were treated under “pure local anesthesia” and found improvements in IPSS (−43.8%), Qmax (+92.3%), and IIEF (+16.7%). The dynamics of IPSS and Qmax changes during follow-up across studies are presented in Figure 3.

### iTIND

Three studies investigated the iTIND procedure for BPH, with 1 RCT and 2 being multi-centric, with follow-up up to 3 years. A total of 241 patients were reported with individual studies ranging from 32 to 128 patients. Patients with a median lobe and prostate volume above 75 mL were excluded. Across studies, the mean baseline IPSS varied from 19.0 to 22.5 and the mean Qmax varied from 7.3 to 8.7 mL/s. The use of LA was reported in 2 studies, with 1 reporting it in all patients (Table 3). Two studies separately indicated the rate of complications, being up to 9.9%, and they were all associated with acute urinary retention (AUR). The retreatment rate varied from 0% to 8.7%. Significant improvement in sexual outcomes was found in 1 study (Table 4).

**Figure 4.**
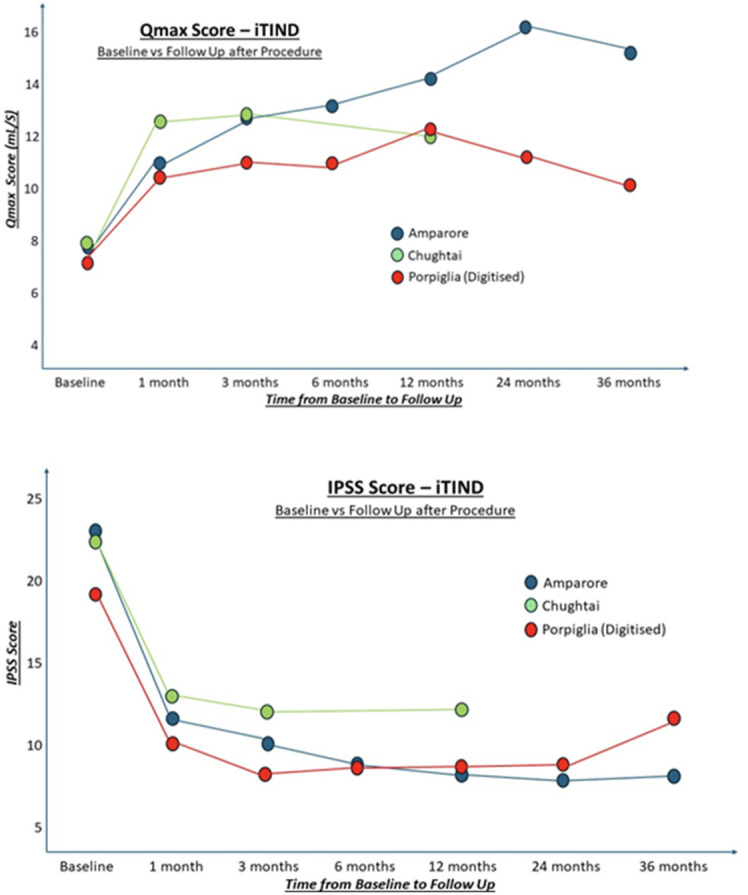
iTIND IPSS and Qmax across studies.

Porpiglia et al^22^ performed a prospective, single-centre cohort study to follow the outcomes of 32 patients who were treated with iTIND and revealed improvements in IPSS (−19%) and Qmax (+41%). Amparore et al^23^ found improvements in IPSS (−58.2%) and Qmax (+114.7%). Chughtai et al^24^ included 138 patients from 9 centers and found the procedure with improvements in IPSS (−42.7%), Qmax (+41.8%), and IIEF (+11.3%). The dynamics of IPSS and Qmax changes during follow-up across studies are presented in Figure 4.

## Discussion

The studies included in this review each examined the detailed outcomes of MIST for male LUTS, secondary to BPH, which can be performed in an office or bedside environment under LA. All studies included had a minimum follow-up of 12 months, allowing for a comprehensive assessment with long-term risks.^25^ They all were done in an office-based setting and used LA to a variable degree.

For the Rezum cohort, there was a consistent improvement seen in Qmax scores across all 6 studies, with a mean improvement in Qmax score of +54.24% from the respective baselines. The largest improvement of 76.5% was seen by Cindolo et al^15^ (*P* < .001). Similarly, Elterman et al^16^ found that there was a 73.9% increase in Qmax after 12 months. Bausch et al^14^ reported on Qmax up to and beyond 2 years, with a mean increase of 66.7%. The paper by McVary et al^17^ exhibits a considerable improvement in Qmax outcomes in the first 3 months after treatment, followed by a steady decline until the final recording at 36 months with a final improvement of +39% (*P* < .0001).

For the UroLift procedure, the outcomes saw a reliable improvement in Qmax scores. Overall, there was a mean percentage increase in the Qmax score of +58.35%. The largest improvement was measured by Secco et al^21^ at 12 months, where a total improvement of +92.3% (*P* < .0001) was seen. Interestingly, this patient population had the lowest average baseline recording for both Qmax and symptom scores, showing that this procedure was highly effective in functionally improving their flow. The RCT by Roehrborn et al^19^ shows a 53.1% (*P* < .0001) improvement at final recording, 36 months after initial treatment.^19^ When examining Figure 3, it does appear that the treatment outcomes of treatment are durable across all 4 papers that reviewed the UroLift procedure.

For the iTIND procedure, the outcomes saw a swift improvement in Qmax after 1 month, followed by mixed outcome over time (Figure 4). When collated, the mean improvement in Qmax at the final outcome recordings for the 3 papers was +65.8%. The largest average increase in Qmax outcomes was seen by Amparore et al^23^ who saw data improve steadily to peak at 24 months and then drop slightly at 36 months to give a final improvement of +114.7% (*P* < .0001). In contrast, Porpiglia et al,^22^ with the same 36-month follow-up time, saw a lesser improvement of +41.0% (*P* < .05) as well as a steady decline of Qmax after the 12-month follow-up. This outcome is similar to that seen in the RCT by Chughtai et al^24^ who had a final mean improvement of +41.8%. The data from this paper is arguably the most significant due to both the study design and the sample, with increased statistical power and minimizing statistical outliers.^26^

While the efficacy of a treatment intervention may be related to various factors, including Qmax improvement, it is essential to measure symptom scores to elicit a personal and subjective measurement of this impact.^27^ In Rezum, there was a clear and sustained improvement in IPSS across the range of papers, with an average improvement of −60.4%. The largest improvement in IPSS was measured at −73.9% (*P* < .001) by Elterman et al.^16^ When considering the durability of outcomes, the RCT by McVary et al^17^ showed significant improvement (−50.0%) in IPSS measured over 36 months, and this remained stable from 3 months onward. It was proposed that there exists a significant inverse correlation between IPSS and Qmax,^28^ yet in this review, it is notable that despite a steady decline in Qmax scores from 3 months after treatment and beyond, there is no coincidental rise in IPSS score.^17^

In the UroLift group, a good improvement in IPSS was measured across each of the 4 studies, with an average improvement in mean IPSS score of −43.3%. The largest of these changes was from Sievert et al^20^ who saw a sustained −51.15% (*P* < .0001) improvement in IPSS from baseline to 24 months post-treatment. Similar plotted data is seen in the review by Secco et al.^21^ The RCT by Roehrborn et al^19^ had the longest follow-up of 36 months and this data showed maximum symptom improvement 3 months after treatment and then a steady increase in scoring over time to finally see a −41.1% improvement at 36-month follow-up. However, there was significant attrition bias in the study.^29^

In the iTIND group, like UroLift and Rezum, a pronounced improvement in IPSS, followed by a reasonable durability are noted. At final recording across the 3 papers, there is a mean improvement in IPSS of −39.9%. A large mean IPSS improvement of −58.2% (*P* < .0001) was seen in the study by Amparore et al.^23^ Porpiglia et al^22^ do see a slight IPSS deterioration after 24 months post-treatment.

In terms of sexual function, there were no significant negative or positive changes to sexual outcomes recorded across any of the 13 papers in this review. Authors generally suggested that this is because sexual outcomes are not of a primary concern for their aging patients, so these parameters were rarely examined.^14^ Despite this, across the various studies, there is generally a consensus that sexual function is unaffected as a result of these minimally invasive treatments. On comparison, in a meta-analyses, TURP can lead to a 65% ejaculatory dysfunction.^30^ This finding then emphasises that maintenance of sexual outcomes is one of the primary benefits which MISTs like Rezum, UroLift, and iTIND offer over invasive procedures such as TURP.

This study had 10 observational cohort studies, and it gave the ability to monitor the incidence of rare and ‘incidental’ adverse events which may be caused by a certain procedure.^31^ Across studies, that there were some minor adverse events (AEs) (Clavien-Dindo I or II) which were consistently reported shortly after treatment. All papers saw some incidence of haematuria and dysuria. The study by Babar et al^13^ recorded that nearly 66% of patients experienced ‘gross haematuria’ following the Rezum procedure, although the average rates in other studies were around 15.4%. Similarly, penile burning was recorded in 64.5%; this was defined as ‘any irritation in the penis after the procedure’. There were rarely any serious complications (Clavien-Dindo III-IV) associated with the procedures. Acute urinary retention was the most frequently reported perioperative complication which was classified as more serious as it required immediate intervention. This was reported by Cindolo et al^15^ at 13.4%; Cantwell et al^18^ at 1.9%; Amparore et al^23^ at 9.9% and Chughtai et al^24^ at 1.7%.

Of the 3 MISTs reviewed, the UroLift group had the highest rate of retreatment (6.4%) at final follow-up. A complication specific to UroLift was encrustation of implants. In the study by Cantwell et al,^18^ a total of 10 implants were recorded to display encrustation due to incorrect placement of implant, leading to exposure to urine after treatment. When more broadly considering the limitations of these 3 MISTs, 1 clear drawback of the UroLift procedure is the use of the permanent implant. Encrustation is a consequence of implantation being performed too close to the bladder neck and that this risk is minimized when proper technique is employed.^32^

Operative time is one of the necessary considerations when examining safety in a procedure.^33^ For the Rezum procedure, the average treatment length was 8.9 minutes, with the shortest Rezum procedure length being 4.8 minutes from Elterman et al.^16^ In the iTIND cohort, the average procedure time was only measured by Porpiglia et al.^22^ They found that the device implantation procedure lasted an average of 5.8 minutes while the removal procedure was quicker and only lasted 2.8 minutes, on average. Finally, for the UroLift procedure, all 4 papers published results on the average procedure time. When collated, the average length of procedure was 48 minutes. While this UroLift treatment might appear less attractive in terms of length of procedure, it should be mentioned that all these are performed in a bedside environment. This method of treatment expedites the recovery process and allows for patients to arrive in a clinic, receive treatment and be eligible to leave on the same day.^34^

Some of the studies included were observational studies, and there is always an issue of recall bias, and data may not be as reliable as they are reliant on patient’s memory of their symptoms.^35^,^36^ Comparisons are sometimes difficult due to the variable duration of follow-ups in these studies.^37^ Regarding MIST procedures, for the sexual function, one of the main focus is ejaculatory prevention.^38^,^39^ This should be compared in future studies. Recent study of Manufacturer and User Facility Device Experience (MAUDE) data on MIST procedures reported on 692 device related complications, and while most were minor, this should also be compared and addressed in future studies.^40^ A second crucial aspect of evaluating any new therapy, including MIST procedures, is the need for long-term follow-up to assess durability and late complications. Amparore et al^41^ have demonstrated a 4-year follow-up for iTIND, setting an important benchmark that all MIST therapies should strive to meet in order to establish their long-term efficacy and safety.

The main limitation of this review is attrition bias,^42^ as dropouts can be due to patient recall, complications or worsening of symptoms. Also, as this study uses MIST under LA, other studies which might be office-based procedures but done only under GA were excluded. Perhaps longer follow-up would be helpful especially in relation to retreatment rates.^43^ Future studies should also look at other MIST procedures such as prostate artery embolisation and Transperineal laser ablation of prostate,^44^ ^45^ and RCTs with multiple treatment options will be helpful in accurate comparisons of outcomes.^46^

## Conclusion

The newer MIST procedures such as Rezum, UroLift, and iTIND can be done safely and successfully as a day case procedure usually under local anesthetic. The short-term results are good for all of these procedures and improves objective outcomes as well as preserves or improves sexual function. However, more randomized trials with multiple options need to be conducted, with published long-term results, which can help patient counselling and decision making.

## Figures and Tables

**Table 1.** Quality Assessment for Observational Cohort Studies

		Did the Study Address a Clearly Focused Issue?	Was the Cohort Recruited in an Acceptable Way?	Was the Exposure Accurately Measured to Minimize Bias?	Was the Outcome Accurately Measured to Minimize Bias?	Have the Authors Identified All Important Confounding Factors?	Have They Taken Account of the Confounding Factors in the Design and/or Analysis?	Was the Follow-up of Subjects Complete Enough?	Was the Follow-up of Subjects Long Enough?
1	Babar et al (2023)	Yes	Yes	Yes	Cannot Tell	Cannot Tell	Cannot Tell	Yes	No
2	Bausch et al (2023)	Yes	Yes	Yes	N/A	No	Yes	Cannot Tell	Yes
3	Cindolo et al (2023)	Yes	Yes	Yes	Cannot Tell	Cannot Tell	Cannot Tell	Yes	No
4	Dixon et al (2016)	Yes	Yes	Yes	Cannot Tell	Cannot Tell	Cannot Tell	Yes	Cannot Tell
5	Elterman et al (2023)	Yes	Cannot Tell	Yes	Cannot Tell	Cannot Tell	Cannot Tell	Cannot Tell	No
6	Cantwell et al (2013)	Yes	Yes	Yes	Yes		Yes	Yes	No
7	Secco et al (2022)	Yes	Cannot Tell	Cannot Tell	Cannot Tell	Cannot Tell	Cannot Tell	No	No
8	Sievert et al (2018)	Yes	Cannot Tell	Yes	Cannot Tell	Cannot Tell	Cannot Tell	Cannot Tell	Cannot Tell
9	Amparore et al (2021)	Yes	Yes	Yes	Cannot Tell	Cannot Tell	Cannot Tell	Yes	Yes
10	Porpiglia et al (2018)	Yes		Yes				Yes	

**Table 2.** Quality Assessment for Randomized Controlled Trials. Critical Appraisal Skills Programme (CASP) (randomized controlled trial)

	McVary et al (2018)	Roehrborn et al (2015)	Chughtai et al (2021)
Did the study address a clearly focused research question?	Yes	Yes	Yes
Was the assignment of participants to interventions randomized?	Yes	Yes	Yes
Were all participants who entered the study accounted for at its conclusion?	Yes	Yes	Yes
Were the participants ‘blind’ to intervention they were given?	Yes	Yes	Yes
Were the investigators ‘blind’ to the intervention they were giving to participants?	Yes	Yes	Yes
Were the study groups similar at the start of the randomized controlled trial?	Yes	Yes	Yes
Apart from the experimental intervention, did each study group receive the same level of care (i.e., were they treated equally)?	Yes	Yes	Yes
Were the effects of intervention reported comprehensively?	Yes	Yes	Yes
Was the precision of the estimate of the intervention or treatment effect reported?	Cannot Tell	Cannot Tell	Cannot Tell
Do the benefits of the experimental intervention outweigh the harms and costs?	Cannot Tell	Cannot Tell	Cannot Tell
Can the results be applied to your local population/in your context?	N/A	N/A	N/A
Would the experimental intervention provide greater value to the people in your care than any of the existing interventions?	N/A	N/A	N/A

**Table 3.** General Characteristics of Included Studies

Authors	Year	Setting	Follow-Up Duration	Number of Patients	Maximum Prostate Volume, mL	Median Lobe	Baseline Characteristics	Percentage of Local Anesthetic Use
**Rezum**								
9. Dixon et al^1^	2016	Multi-centre, prospective	2	65	110	Included	IPSS: 21.6 (13.0-35.0) Qmax (mL/s): 7.9 (1.4-15.0)	n/a
10. Babar et al^2^	2023	Single centre, retrospective	1	238	80	Included	IPSS: 18.0 (11.0-24.0) Baseline Qmax(mL/s): 10.4 (7.8-13.5)	17.6
11. Bausch et al^3^	2023	Single centre, retrospective cohort	2	211	80	Included	IPSS: 18.0 (13.0-23.0) Qmax(mL/s): 8.4 (6.0-12.0)	11.9
12. Cindolo et al^4^	2023	Multi-centre, retrospective cohort	1	352	75	Included	IPSS: 22.0 (18.0-26.0) Qmax(mL/s): 8.4 (6.7-11.0)	n/a
13. Elterman et al^5^	2023	Multi-centre, prospective cohort	1	229	160	Included	IPSS: 22.7 (N/A) Qmax(mL/s): 8.5 (N/A)	19.2
14. McVary et al^6^	2023	Multi-centre, randomized controlled trial	3	197	80	Included	IPSS: 22.0 (+/-4.8) Qmax(mL/s): 9.9 (+/-2.2)	20.7
**UroLift**								
Cantwell et al^7^	2013	Multi-centre; prospective crossover cohort study	1	53	80	Excluded	IPSS: 23.3 (13.0-34.0) Baseline Qmax(mL/s): 8.8 (2.0-30.0)	96
Roehrborn et al^8^	2015	Multi-centre, randomized, blinded controlled trial	3	137	80	Excluded	IPSS: 22.32 (13.0-35.0) Baseline Qmax(mL/s): 7.88 (3.0-13.0)	99.4
Sievert et al^9^	2018	Multi-centre, prospective cohort	2	86	111	Excluded	IPSS: 20.82 (+/-6.52) Qmax (mL/s): 11.24 (+/-3.16)	36.9
Secco et al^10^	2022	Multi-centre, prospective cohort	1	55	90	Excluded	IPSS: 23.8 (+/-4.3) Baseline Qmax(mL/s): 6.8 (+/-2.3)	100
**iTIND **								
Porpiglia et al^11^	2018	Single-centre, prospective cohort	3	32	60	Excluded	IPSS: 19.0 (14.0-23.0) Qmax (mL/s): 7.6 (+/-2.2)	n/a
Amparore et al^12^	2020	Multi-centre; prospective cohort study	3	81	75	Excluded	IPSS: 22.5 (+/-5.6) Qmax(mL/s): 7.3 (+/-2.6)	100
Chughtai et al^13^	2021	Multi-centre, randomized, single-blinded controlled trial	1	128	75	Excluded	IPSS: 22.1 (+/-5.6) Qmax(mL/s): 8.7 (+/-3.3)	27.1

**Table 4.** Reported Outcomes in Included Studies

Authors	Year	Average Procedure Length (min)	Qmax Change	IPSS Change	Sexual Outcomes	Complication Rates	Retreatment Rate
**Rezum**							
Dixon et al^1^	2016	n/a	+44.6%	−55.7%	No clinically significant change	(Clavien-Dindo I or II) included: Urinary retention (33.8%) Dysuria (21.5%) Urgency (20%) Haematuria (13.8%) Nocturia (7.7%) Serious complications (Clavien-Dindo III or IV) included: TURP re-operation due to obstructive median lobe causing poor stream, retention and frequency	1.5%
Babar et al^2^	2023	n/a	+ 24.76%	−44.44%	n/a	(Clavien-Dindo I or II) included: Gross haematuria (66.5%) Penile burning (64.5%) Penile pain (37.1%) Sloughing (23.5%) Urinary retention (13.4%) UTI (6.1%) Serious complications (Clavien-Dindo III or IV) included: Postoperative vasovagal syncope (4 patients – 1.7%)	3.4%
Bausch et al^3^	2023	10.0 (7.0-16.0)	+66.7%	−65.7%	n/a	(Clavien-Dindo I or II) occurred in 25 patients (11.8%): N/A No serious complications (Clavien-Dindo III or IV) were recorded.	5.7%
Cindolo et al^4^	2023	12.0 (9.0-15.0)	+76.5%	−72.7%	+25%	(Clavien-Dindo I or II) occurred in 176 patients (50%): Dysuria (19.6%) Mild haematuria (11.4%) Urgency and burning symptoms (3.2%) Fever—suspected UTI (7.1%) AUR after first catheter removal (13.4%) No serious complications (Clavien-Dindo III or IV) were recorded.	2.5%
Elterman et al^5^	2023	4.8 (1.5-14.0)	+73.9%	−73.9%	No statistically significant change	No serious complications (Clavien-Dindo III or IV) were recorded.	4.4%.
McVary et al^6^	2023	n/a	+39%	−50.0%	−3%	(Clavien-Dindo I or II) included: Dysuria (16.9%) Haematuria (11.8%) Frequency/urgency (5.9%) AUR (3.7%) Acute UTI (3.7%) Serious complications (Clavien-Dindo III or IV) included: N/A	4.4%.
**Urolift**							
Cantwell et al^7^	2014	53.0 ± 15	+35%	37.0%	No change	(Clavien-Dindo I or II) included: Dysuria (36%) Haematuria (26%) Pain/discomfort (21%) Serious complications (Clavien-Dindo III or IV) included: AUR with re-presentation to the hospital (1%)	2%
Roehrborn et al^8^	2015	66.14 (24.0-162.0)	+53.1%	−41.1%	No change	(Clavien-Dindo I or II) included: Dysuria Haematuria Pain/discomfort Urge / urge incontinence No serious complications (Clavien-Dindo III or IV) were recorded.	10.7%.
Sievert et al^9^	2018	57.0 (42.0-90.0)	+53.1%	−51.15%	No change	(Clavien-Dindo I or II) included: Dysuria / Haematuria (14%) Pain/discomfort (3.5%) No serious complications (Clavien-Dindo III or IV) were recorded.	12.8%.
Secco et al^10^	2022	16.0 (8.0-60.0)	+92.3%	−43.8%	+16.7%	31.3% of patients required catheterisation due to haematuria or AUR. No serious complications (Clavien-Dindo III or IV) were recorded.	0%
**iTIND **							
Porpiglia et al^11^	2018	Implantation (sd) = 5.8 (2.5) Removal (sd) = 2.0 (1.0)	+41.0%	−19.0%	No change	4 patient complications (12.5%)	0%
Amparore et al^12^	2020	n/a	+114.7%	58.2%	No change	(Clavien-Dindo I or II) included: Haematuria (12.3%) Dysuria (7.4%) Urgency (11.1%) Pain (9.9%) UTI (6.2%) Serious complications (Clavien-Dindo III or IV) included: AUR (9.9%)	8.7%
Chughtai et al^13^	2021	n/a	+41.8%	42.7%	+11.3%	(Clavien-Dindo I or II) included: Haematuria (13.6%) Dysuria (22.9%) Urgency (5.1%) UTI (1.7%) Serious complications (Clavien-Dindo III or IV) included: AUR (1.7%) UTI (1.7) Sepsis (0.8%)	4.7%

AUR, acute urinary retention; UTI, Urinary tract infection.

## Data Availability

The data that support the findings of this study are available on request from the corresponding author.
